# A novel LMNA mutation (R189W) in familial dilated cardiomyopathy: evidence for a 'hot spot' region at exon 3: a case report

**DOI:** 10.1186/1476-7120-8-9

**Published:** 2010-03-22

**Authors:** Nicoletta Botto, Simona Vittorini, Maria Giovanna Colombo, Andrea Biagini, Umberto Paradossi, Giovanni Aquaro, Maria Grazia Andreassi

**Affiliations:** 1 Genetics Research Unit, Fondazione G Monasterio CNR-Regione Toscana, via Aurelia Sud, Massa, Italy; 2 CNR-Institute of Clinical Physiology, via Moruzzi 1, Pisa, Italy

## Abstract

We describe a case of a patient with idiopathic dilated cardiomyopathy and cardiac conduction abnormalities who presented a strong family history of sudden cardiac death. Genetic screening of lamin A/C gene revealed in proband the presence of a novel missense mutation (R189W), near the most prevalent lamin A/C mutation (R190W), suggesting a "hot spot" region at exon 3.

## Introduction

Lamin A/C gene (*LMNA*) mutations have been causally linked to dilated cardiomyopathy (DCM), accounting for 6% to 8% of all idiopathic DCM up to 40% when conduction disorders are present [1,2]. In particular, *LMNA*-related DCM is characterized by progressive heart failure, variable involvement of skeletal muscle, atrioventricular block and/or tachyarrhythmias [2]. Furthermore, *LMNA *mutations have been shown to be associated to a very poor prognosis, with a high rate of sudden cardiac death, and severe forms of heart failure necessitating heart transplantation [3,4].

We report a case report of DCM with a family history of sudden cardiac death (3 brothers dead for sudden cardiac death) in which a novel *LMNA *missense mutation was found. In addition, we discuss the role of genetic testing for the *LMNA *gene in routine clinical practice.

## Case presentation

The proband was a 55 year-old woman referred to our Cardiology Department for fatigue, palpitations and cardiac dyspnea at rest (NYHA Class IIa). LV function assessed by echocardiography showed an ejection fraction of 36% with dilatation of the left ventricle (end-diastolic diameter 59 mm) and a wall motion score index of 2 (global hypokinesia). During a Holter recording the patient presented premature ventricular beats with an incidence rate of 5 per hour. No complex tachyarrhythmias were observed (Table 1). DCM was diagnosed on the basis of Word Health Organization criteria [5]. Moreover, the patient underwent stress echocardiography that showed contractile reserve with normalization of function at peak stress.

**Table 1:** Baseline characteristics of the proband.

**Onset age (years)**	55
**Gender**	F
**NYHA**	IIa
**Electrocardiogram**	Premature ventricular beats
**LVEF (%)**	36
**LVEDV (cm^3^)**	157
**LVEDD (mm)**	59
**LVFS (%)**	17
**sCPK (mU/ml)**	70
**Mutation other than missense**	861 t>c (A287A) exon 5
	1338 t>c (D446D) exon 7
	1698 c>t (H566H) exon 10

The proband was prescribed ACE-inhibitors and is followed-up at our out-patient clinic. At last visit (8 years after first observation) the patient is asymptomatic for dyspnea (NYHA Class I) and/or palpitations. The echocardiogram shows a recovery of LV function with an ejection fraction of 57% and sporadic premature ventricular beats at Holter recording.

**Figure 1:**
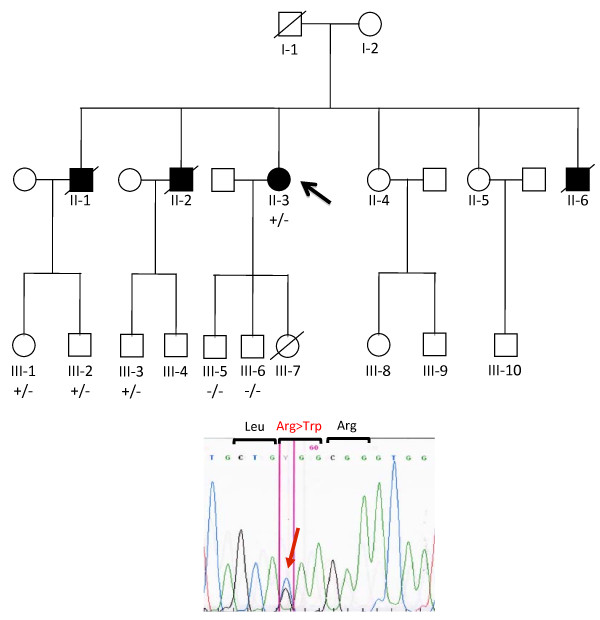
**Pedigree of the family**. Individuals are indicated by generation and pedigree number. The proband is indicated by arrow and affected status is indicated by filled symbol. The presence (+) or absence (-) of the mutation is indicated for the genetically tested family members. Chromatogram below demonstrates the heterozygous mutation.

Family history revealed that three proband's brothers died of sudden cardiac death at the age of 72, 44 and 22 years, respectively (Figure 1). After genetic counselling, on the basis of clinical data and family history, the patient underwent to genetic screening of *LMNA *gene mutations.

### Genetic testing

Genetic testing was performed in the proband by screening of the 12 coding exons of *LMNA *gene amplified by polymerase chain reaction (PCR) using primers encompassing the protein coding region of exons as well as the immediate intronic regions essential for splicing, as described previously [6]. Sequencing was performed with a capillary sequencer CEQ 8800 (*Beckman Coulter, Germany*) according to the manufacturer's protocol.

**Figure 2:**
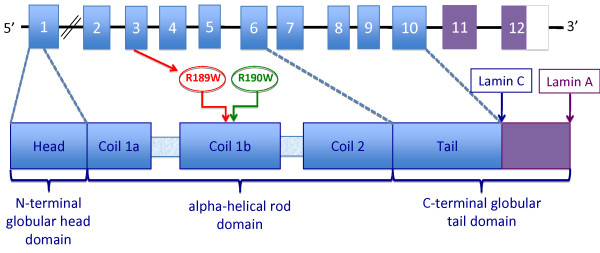
**Localization of the R189W mutation in lamin A/C gene and lamin A/C protein**. The mutation is located in exon 3 coding for coil 1b in alpha-helical rod domain.

A novel heterozygous missense mutation cytosine-to-thymine at nucleotide 565 in exon 3 was identified in the proband. The nucleotide change predicts an arginine (basic) to tryptophan (hydrophobic) substitution (R189W) in a conserved residue located in the coil 1b of the alpha-helical rod domain (Figure 2) (*GenBank accession n*. NM_170707.2).

The mutation was confirmed by PCR-based restriction fragment length polymorphism (PCR-RFLP) analysis by using specific primers (forward 5'-GCAGCAGCCCACCTCTC-3'and reverse 5'-AAGGCGAGCTCTGCACAC-3') and the restriction enzyme *Tau I *that cut the wild-type sequence into two fragments of 167 bp and 134 bp. In the presence of 565c>t transition, *Tau I *does not recognize the restriction site and leaves the 301 bp PCR product uncutted.

The R189W mutation was neither identified in 100 chromosomes from 50 normal volunteers (≥ 30 years of age) who were randomly selected from our control genomic store, nor previously published in the *LMNA *literature as benign polymorphism, indicating it is likely not a common variant. A total of three synonymous substitutions were also found in the proband, but none of these nucleotide changes predicts a change in aminoacid sequence (Table 1). These 3 silent variants were already described and registered in the databases and have no conclusive or known pathogenicity.

No genetic material or medical records were available for parents and 2 sisters of the proband. After giving informed consent, the other family members received genetic counselling and clinical screening, and underwent peripheral blood sampling for genetic testing.

Clinical investigation included resting cardiologic evaluation, 12-lead electrocardiogram and 2-dimensional echocardiography study. During genetic screening, the specific R189W mutation was identified in three asymptomatic nephews (III-1, 37 years; III-2, 31 years and III-3, 32 years) and excluded in the two sons of the proband (III-5 and III-6). The mutation carriers III-1, III-2, and III-3 were asymptomatic and presented normal ECG.

**Figure 3:**
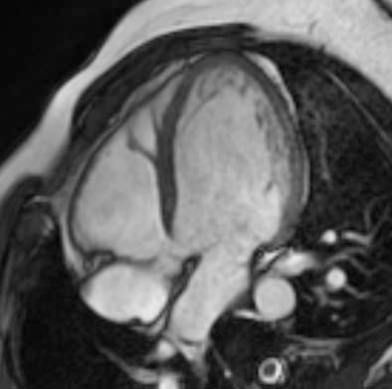
**Cardiac magnetic resonance of III-10 subject showing a sponge-like appearance and trabeculated fibres in the lateral wall**.

One nephew (III-10, 30 years) underwent magnetic resonance testing for the confirm of non-compaction cardiomyopathy suspected at echocardiography. Magnetic resonance imaging showed a sponge-like appearance and trabeculated fibres in the lateral wall, but did not fulfil the diagnostic criteria for ventricular non-compaction cardiomyopathy (Figure 3). The subject is unavailable for genetic screening at the moment.

## Discussion

Our report describes a family in which proband and clinically unaffected family members harbour a novel potentially pathogenic mutation (R189W) in *LMNA *gene.

At the present, *LMNA *gene is the most frequent disease-associated gene for familial DCM with conduction system disease. The *LMNA *gene encodes the two differentially spliced proteins lamin A and lamin C, the major components of the nuclear lamina, which localizes at the nucleoplasmic surface of the inner nuclear membrane as a meshwork structure [2]. Lamin interacts directly with the chromatin and also with the integral proteins of the inner nuclear membrane, thereby playing a role in maintaining the structural integrity and spatial organization of other inner nuclear membrane.

It is worth to mention that the most prevalent *LMNA *mutation "hot spot" in familial DCM is codon 190 in exon 3. The mutation R190W was initially described by Arbustini et al. in an Italian family with severe DCM and sudden cardiac death, and subsequently found in another family in Europe and other countries associated with conduction system disease and an ominous outcome [1,7-9]. Codon 190 is localized in the protein domain coil 1b, an alphahelical segment of the central rod separated by short linkers, which is important for lamin B interaction and lamin A/C dimerization. Mutations in this domain, disrupting the coiled-coil fold of lamin A and C, may lead to a weaker nuclear lamina with subsequent mechanical stress damage by muscle contraction and abnormal mechano-transduction [10]. Our novel mutation is located in the previous codon 189, leading to the same aminoacid substitution (R189W), suggesting a "hot spot" region at exon 3 (Figure 2).

*LMNA *is not only the most frequent gene found in DCM, but it has also been shown to be associated with a very poor prognosis and a high mortality rate [3,4]. A meta-analysis showed that *LMNA *mutations carriers are at very high risk for arrhythmogenic complications, with a class I indication to defibrillator implant. Most cases, in fact, experience sudden cardiac death and show malignant course with severe forms of heart failure necessitating heart transplantation [3]. Recently, Pasotti et al. have shown that dilated cardiomyopathies caused by *LMNA *gene defects are highly penetrant, have adult onset, are malignant conditions characterized by a high rate of severe left ventricular dysfunction and life-threatening arrhythmias, that should lead to considering special indications for ICD implantation in this group of patients [4].

Therefore, the severity of *LMNA *mutations strengthens the need for genetic testing of *LMNA *in order to risk stratify DCM patients and their relatives, and indicate an early treatment so to avoid the main complications of the disease [2,11].

Genetic counselling is becoming a mainstay in the clinical management of familial diseases. It is important to appropriately select the patients (and families) to be screened in order to identify the subset that should be followed-up and treated accordingly.

Genetic testing is often the best way to provide risk estimates for asymptomatic patients. However, genetic tests remain generally expensive technologies that are labor-intensive and time-consuming. Therefore, routine and extensive genetic screening is impractical because of the genetic heterogeneity of DCM. Genetic testing is not appropriate for every patient, but it should be used in selected cases, such as patients with an established family history of severely affected relatives and at high risk of worse prognosis.

In particular, our results strengthen the evidence that genetic screening is indicating in idiopathic DCM with a positive family history in ≥ 2 closely related relatives [2]. These cases generally show an autosomal dominant pattern of inheritance and have underlying mutations in specific genes; some of these mutations are known to be associated with a more malignant phenotype.

Genetic testing unambiguously allows for early identification and diagnosis of individuals at greatest risk for developing DCM, allowing to focus clinical resources on high-risk family members. In addition, it is extremely important that family members receive careful counselling before and after testing on the potential risks.

Relative may carry the mutation but be asymptomatic, and the mutation may merely be a predisposing factor to disease in the presence of other factors, and so its presence alone does not allow accurate prediction of phenotype or prognosis. However, if a mutation is identified in asymptomatic individual, regular clinical cardiovascular screening (echocardiogram, ECG) is recommended to detect the first signs of disease that may be diminished by early treatment. If family members are found not to carry that mutation, they can be definitively diagnosed as unaffected, and the need for serial follow-up becomes unnecessary. In this case, they can be reassured that neither they nor their offspring will be at higher risk compared to the general population to develop these disorders.

The identification of the R189W mutation in our proband, led us to recognize three non-affected mutated carriers at higher risk of DCM who will benefit from regular clinical cardiac follow-up (echocardiogram, ECG) and early treatment. On the other hand, the exclusion of the causal mutation in two sons of the patient enables termination of the periodic clinical screening, allowing to address clinical resources only on high-risk family subjects.

Genetic testing is, therefore, clinically useful and should be recommended for DCM families.

## Consent

Written informed consent was obtained from the patient for publication of this case report and any accompanying images. A copy of the written consent is available for review by the Editor-in-Chief of this journal.

## Competing interests

The authors declare that they have no competing interests.

## Authors' contributions

NB carried out the molecular genetic studies and wrote the manuscript; SV and MGC carried out the molecular genetic studies and have been involved in revising it critically for important intellectual content; GA performed cardiac MRI and has been involved in revising of the manuscript; UP and AB performed the clinical assessment of the subjects and has been involved in revising of the manuscript; MGA defined the study design and wrote the manuscript.

All authors read and approved the final manuscript.
